# Effects of barley green on uric acid, inflammatory factors, xanthine oxidase activity and body composition of patients with hyperuricemia: a randomized controlled trial

**DOI:** 10.3389/fnut.2025.1684829

**Published:** 2025-10-31

**Authors:** Mingxuan Cui, Lin Shao, Shilong Zhao, Qianying Guo, Xinran Liu, Peng Liu

**Affiliations:** Department of Clinical Nutrition, Peking University People’s Hospital, Beijing, China

**Keywords:** barley green, uric acid, inflammatory factors, xanthine oxidase activity, body composition

## Abstract

**Aim:**

This study aims to evaluate the efficacy of barley green intervention in reducing uric acid (UA) levels, as well as its effects on related inflammatory factors, metabolic indices and body composition in individuals with hyperuricemia. Additionally, we investigate the mechanism of action associated with xanthine oxidase (XOD).

**Materials and methods:**

In this randomized controlled trial, patients diagnosed with hyperuricemia were randomly assigned to either an intervention group receiving barley green alongside a balanced diet regimen or a control group adhering solely to a balanced diet. Comprehensive clinical data were collected from participants, and nutritional measurements, UA metabolism-related indicators, inflammatory factor levels, and XOD activity were assessed at various stages over a 3-month period.

**Results:**

A total of 90 subjects participated in the study; 49 individuals were included in the intervention group while 25 comprised the control group. The reduction in UA levels-the primary endpoint-was significantly greater after 3 months of intervention compared to those following only a balanced diet [55.00 ± 62.39 vs. 25.00 ± 59.52 (*p* = 0.049)]. Improvements in XOD activity and body composition were also observed within the intervention group relative to controls. Multiple linear regression analysis indicated that baseline UA levels, XOD activity, leptin concentrations, as well as changes in total cholesterol and body weight were independently associated with the reduction in UA following a 3-month intervention.

**Conclusion:**

Oral administration of barley green may contribute to UA regulation and improvements in body composition among individuals with hyperuricemia, with the reduction in XOD activity potentially serving as one of the underlying mechanisms by which barley green exerts its urate-lowering effect.

**Clinical trial registration:**

ClinicalTrials.gov, identifier NCT06876909.

## Introduction

Hyperuricemia is a chronic disease characterized by elevated uric acid (UA) levels caused by disrupted purine metabolism. It is strongly linked to metabolic disorders such as diabetes, dyslipidemia, obesity, hypertension, arteriosclerosis and coronary heart disease (1). In recent years, its prevalence has risen globally, with increasing incidence among younger individuals. Current treatments mainly include pharmacological therapy, such as allopurinol—a xanthine oxidase inhibitor (XOI)—and medical nutritional therapy (MNT), typically involving a low-purine diet. However, drug therapies may cause serious side effects including fever, allergic rash, abdominal pain, diarrhea, leukopenia and thrombocytopenia, multiple organ damage, and even death (2). Furthermore, studies show that low-purine diet reduce UA levels by only about 30%, and long-term dietary adherence is difficult (3). As a result, there is growing interest in exploring medicinal plants as alternative therapies, which are believed to offer better efficacy and safety than conventional therapies.

Barley green is an extract from young barley leaves, encompassing over 200 nutrients and abundant in phytochemicals such as chlorophyll, plant flavonoids and active enzymes. As a result, it can be considered a complex plant-based nutrition source. Studies show that barley grass has various pharmacological activities, including anti-cancer activity, antioxidative properties, anti-inflammation effects, liver protection, and regulation of glucose and lipid metabolism (4). Animal and observational studies support its potential as a medicinal plant for lowering UA levels by increasing urine alkalinity, reducing inflammation, and regulating metabolism (5, 6).

However, few human clinical trials have investigated UA-lowering effects of barley green through direct consumption. Therefore, it’s clinical efficacy as an UA-lowering agent needs further validation in real-world settings.

We hypothesize that oral administration of barley green may reduce serum UA levels, regulate inflammatory and metabolic markers, and improve body composition in patients with hyperuricemia who follow a balanced diet. It may also reduce UA levels by inhibiting XOD activity.

This randomized controlled trial involved patients with hyperuricemia. The primary objective was to investigate the effect of barley green on UA reduction under a balanced dietary regimen. Secondary outcomes included changes in inflammatory and metabolic indicators and body composition. We also conducted a preliminary assessment of whether barley green lowers UA by inhibiting XOD activity.

## Materials and methods

### Trial design and subjects

This was a single-center, randomized controlled trial.

Patients with hyperuricemia from Peking University People’s Hospital were recruited for this study between July 1, 2021, and December 30, 2023, with follow-up completed by June 30, 2024.

The inclusion criteria were as follows:

Age ranging from 18 to 65 years; under a normal purine diet condition, non-consecutive fasting serum UA levels on two different days: for males, serum UA levels between > 420 μmol/L and < 540 μmol/L; for females, serum UA levels between > 360 μmol/L and < 540 μmol/L; participants must be willing to undergo assessment and provide informed consent.

The exclusion criteria were as follows:

1. Patients currently receiving medication treatment for hyperuricemia.
2. Individuals suffering from diseases that affect food digestion and absorption (such as chronic diarrhea, constipation, severe gastrointestinal inflammation, active gastrointestinal ulcers, post-gastrointestinal resection or cholecystectomy).
3. Individuals with cardiovascular or cerebrovascular diseases, grade III hypertension, chronic hepatitis, malignant tumors, anemia (hemoglobin value below the normal reference range), mental disorders including memory impairments or epilepsy.
4. Subjects concurrently receiving other functional food nutritional support (including plant active substances or health foods).
5. Patients exhibiting abnormal liver function (alanine aminotransferase or aspartate aminotransferase exceeding three times the upper limit of normal) or abnormal renal function (serum creatinine exceeding the upper limit of normal).
6. Those diagnosed with active tuberculosis, AIDS, or other infectious diseases.
7. Individuals with severe allergies to any components of the study nutrition.
8. Pregnant or lactating individuals.
9. Patients with physical disabilities deemed clinically unsuitable for participation in the study (for example: those suffering from severe illnesses not included in the exclusion criteria).
10. Episodes of gouty arthritis occurring two times or more during the specified period.
11. A gouty arthritis attack occurs once, accompanied by serum UA levels exceeding 480 μmol/L, or in conjunction with any of the following criteria: age under 40 years, presence of tophi or evidence of urate deposition within the joint cavity, UA nephrolithiasis or renal dysfunction (eGFR ≤ 89 mL/ (min·1.73m^2^)), hypertension, impaired glucose tolerance or diabetes mellitus, dyslipidemia (any one of the four lipid parameters surpassing the normal reference range), obesity (BMI ≥ 28 kg/m^2^), coronary heart disease, stroke, or heart failure.
12. Serum UA levels greater than 480 μmol/L combined with any of the following conditions: UA nephrolithiasis or renal dysfunction (eGFR ≤ 89 mL/ (min·1.73m^2^)), hypertension, impaired glucose tolerance or diabetes, dyslipidemia (any one of the four lipid parameters exceeding the normal reference range), obesity (BMI ≥ 28 kg/m^2^), coronary heart disease, stroke, or heart failure.

Individuals who meet all the inclusion criteria and do not exhibit any exclusion criteria mentioned above will be enrolled in this study.

### Randomization grouping and study intervention

**Figure 1:**
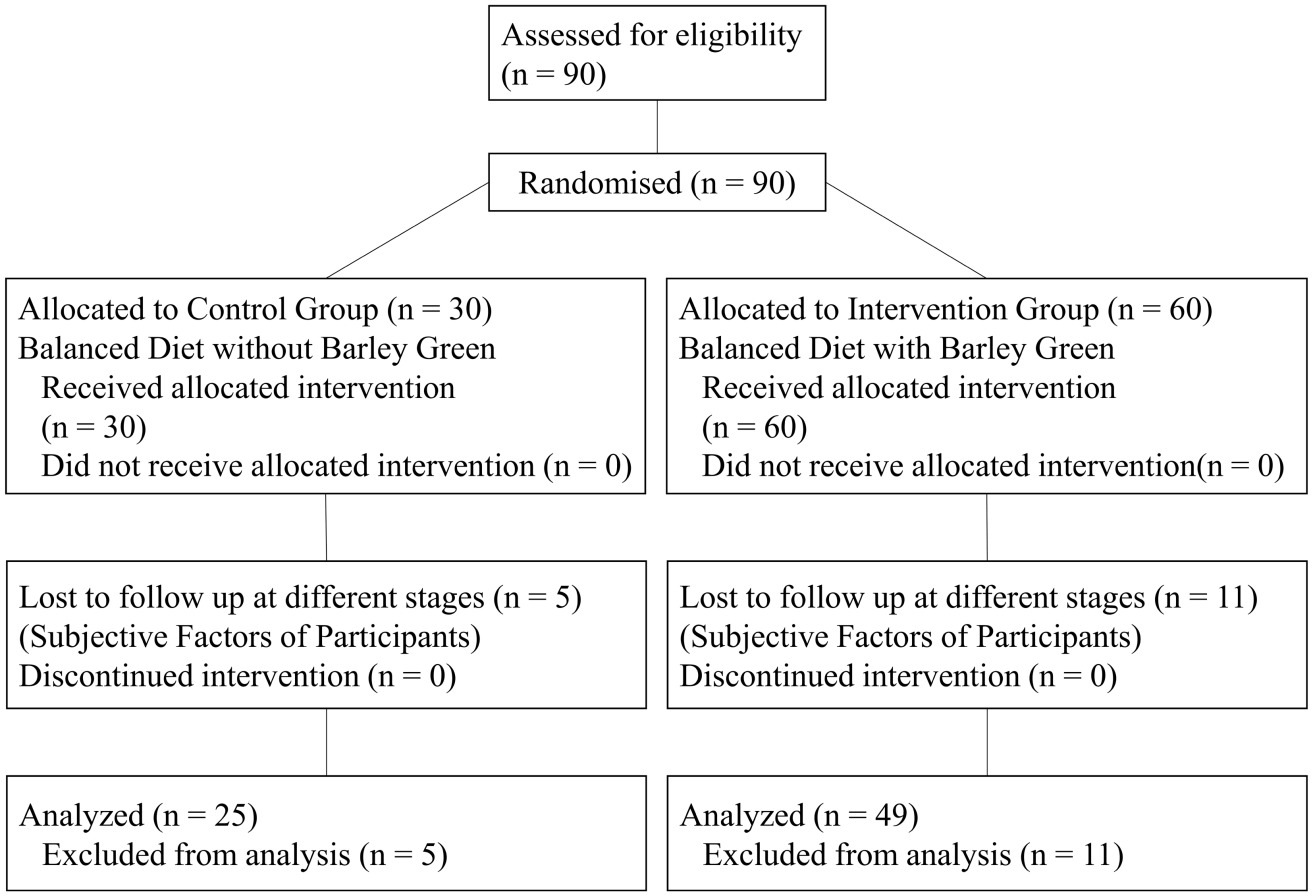
CONSORT flow diagram.

Note: During the research period, subjects who took UA-lowering medications such as allopurinol, febuxostat, or benzbromarone; Chinese herbal medicines with UA-lowering properties; specialized medical formula foods; health supplements; or any foods known to lower UA levels were required to promptly inform the researchers and discontinue participation in the trial.

A completely random design was employed in this study. Following the generation of a random allocation sequence using Microsoft Excel by the principal investigator, doctors and nurses within the research team were tasked with recruiting participants and assigning them to groups based on their order of consultation without knowing of the specific random allocation sequence. The allocation concealment was fully ensured. Eligible participants who met both inclusion and exclusion criteria provided informed consent before being randomized into groups (at a 2:1 ratio) using a designed random number table which is known only to the principal investigator. Participants were randomly assigned to either the intervention group or the control group (see Figure 1). This trial did not incorporate blinding; instead, the control group followed a balanced dietary pattern exclusively. Health education and dietary guidance were delivered in accordance with the Dietary Guidelines for Hyperuricemia and Gout Patients (WS/T560-2017) as well as China Residents’ Balanced Diet Pagoda (2022) on days 1, 30, and 60 of the trial. The recommended daily food intake is illustrated in Supplementary file 1.^1^ The dietary guidance approach for the both groups was identical; however, subjects in the intervention group consumed barley green daily throughout the entire duration of the intervention. The barley green product utilized in this study was Mai Ji Jian Mai Su, which was provided free of charge by Hangzhou BOK Biotechnologies Co., Ltd. (Production License No. (SC10633010606735)). This product consists entirely of 100% barley green- a substance extracted from young green barley leaves at BOK’s planting base, which is located in an area remote from pollution sources and characterized by superior environmental conditions. Mai Ji Jian Mai Su exclusively uses young barley seedlings planted over winter as raw materials. These seedlings have a growth cycle of 60–90 days and are exposed to sunlight for over 800 h. Moreover, advanced biotechnological methods are adopted in the production process, including complete cell wall disruption, hundredfold concentration, and instantaneous vacuum drying at room temperature. The two core technologies, namely “cell wall disruption” that breaks down the plant cell walls impeding human absorption and “ambient-temperature drying” that instantaneously dries the components under vacuum conditions with a surface temperature below 40 degrees Celsius, are independently developed. These technologies have been awarded national invention patents. Participants in the intervention group were instructed to take one packet (4 g) of Mai Ji Jian Mai Su powder along with 10 tablets of Mai Ji Jian Mai Su each time, twice daily—once in the morning and once in the evening—consuming them half an hour prior to meals.

### Clinical data and sample collection, laboratory tests, and endpoints

Nutritional measurement indicators, including height, weight, and body composition (InBody 770), were assessed on the first day of enrollment and subsequently at 30, 60 and 90 days during the trial. Five milliliters of blood were collected for the evaluation of blood counts, urinalysis, erythrocyte sedimentation rate (ESR), clinical indicators related to UA metabolism (comprehensive biochemical panel), as well as serum inflammatory markers such as adiponectin (ADI), leptin and interleukins (IL). The activity of XOD was also measured. Laboratory tests required participants to fast for more than 8 h; venous blood samples were drawn in the early morning following this fasting period. Among these assessments, biochemical indicators—including serum UA—were determined using enzymatic methods. Inflammatory factors were quantified through enzyme-linked immunosorbent assay (ELISA) and flow cytometry (FCM). The activity of XOD in plasma was evaluated utilizing a specific detection kit (Human Xanthine Oxidase ELISA Kit, NBP2-82392). Plasma samples were collected using EDTA or heparin as anticoagulants. The samples were centrifuged at 1000 × g for 15 min at 2–8 °C within 30 min of collection, and the supernatant was obtained. The standard working solution and samples were added in duplicate to microplate wells and incubated at 37 °C for 90 min. The biotinylated detection antibody working solution was then added and incubated for 1 h at 37 °C without washing. After three washes with wash buffer, optionally using a microplate washer, the HRP conjugate working solution was added and incubated for 30 min at 37 °C, followed by five washes. Substrate reagent was added and incubated in the dark for up to 30 min. Finally, the stop solution was added in the same order, and OD values were immediately measured at 450 nm.

The variation in serum UA levels between both groups before and after the intervention served as the primary outcome measure. Secondary outcome measures included alterations in clinical indicators such as body weight, BMI, body composition (including body fat and visceral fat), blood glucose levels, lipid profiles, white blood cell count, ESR; changes in inflammatory factors including ADI, leptin, IL1, IL2, IL4, IL-6, IL10, TNF-*α*; along with variations in XOD activity throughout the intervention. These differences were calculated based on data obtained from measurements taken on enrollment day compared to those taken on the 90th day.

### Visits and follow-up

In addition to regular face-to-face follow-up visits at the nutrition clinic conducted individually at four key time points (the day of enrollment, 30 days, 60 days, and 90 days), the research team—comprising both a dietitian and a nurse— provided nutrition counseling and monitored participants’ dietary habits every 2 to 4 weeks. Participants were asked to maintain dietary records for this purpose. The researchers made timely adjustments to the participants’ dietary habits to promote a balanced nutritional pattern. The compliance with barley green intake was monitored by self-record. Within 1 month following the conclusion of the study, researchers conducted follow-up assessments via phone or in-person visits to determine whether any adverse events related to the intervention foods had occurred.

### Sample size and statistical analysis

The sample size was calculated based on the changes observed in the primary outcome variable, namely the UA level. Drawing from the pre-experimental findings of a multi-center study titled “Observation on the Clinical Effects of Barley Green Intervention in Hyperuricemia Population”(Horizontal Cooperation Project of Zhejiang University, Project number: K Heng 20200443), it was noted that the serum UA levels before and after intervention in the barley green group were 455.50 ± 51.48 μmol/L and 413.17 ± 69.91 μmol/L, respectively. In contrast, for the control group, serum UA levels remained consistent at478.50 ± 56.70 μmol/L and 478.50 ± 57.12 μmol/L before and after the experiment, respectively. With an *α* level set at the 0.05 and a type II error probability (*β*) established at 0.1—resulting in a test power (1−*β*) of 0.9—the sample sizes were estimated using PASS 11 software to be 42 participants for the intervention group and 21 for the control group, respectively. Taking into account a projected loss-to-follow-up rate of approximately 30%, we adjusted our sample sizes to accommodate this potential attrition; thus, each group’s final target size was set at 60 for the barley green intervention group and at least 30 for the control group, culminating in a total required sample size of approximately 90 participants.

Data were presented as mean ± SD, median (interquartile range), or counts (%). Mean alterations from baseline to the conclusion of the study were reported as either mean or median (interquartile ranges). Within-group variations were assessed using paired *t*-tests or the Wilcoxon signed-rank test. Differences between groups were analyzed with independent-sample *t*-test or Mann–Whitney U-test for continuous data, and chi-squared test for categorical data. The difference in UA was normally distributed; therefore, mean ± SD was employed for data description, and independent sample *t*-tests were utilized to analyze intergroup differences. Correlations between changes in UA and other relevant variables were evaluated through Pearson correlation analysis or Spearman rank correlation analysis. Multivariate analyses were conducted using multiple linear regression to longitudinally identify factors associated with the primary outcome. Data analysis was performed using IBM SPSS Statistics 27 and GraphPad Prism 8. A *p*-value of less than 0.05 was considered statistically significant. Missing values were addressed by excluding cases on a test-by-test basis. Outcome evaluators remained unblinded to the assigned interventions.

## Results

### Trial population and baseline clinical characteristics

A total of ninety subjects were randomized into either the intervention group or the control group at an approximate ratio of 2:1. Of these, 74 participants completed the trial, comprising 49 in the intervention group and 25 in the control group. The baseline characteristics are presented in Table 1, and most of the measures demonstrated statistically significant (*p* > 0.05) between the two groups, except for glucose, XOD and leptin (*p* = 0.01, 0.002 and 0.003 separately).

**Table 1:** Demographic and clinical characteristics at baseline.

Variables	Intervention group (*n* = 49)	Control group (*n* = 25)	*p*-value
Age, years*	36.00 (30.00, 43.50)	35.00 (30.00, 43.50)	0.84
Female (%)	21 (42.86)	5 (20.00)	0.05
Male (%)	28 (57.14)	20 (80.00)	0.05
Weight, kg*	76.400 (64.275, 99.550)	86.000 (78.750, 109.600)	0.23
BMI, kg/m^2^	29.15 ± 4.74	29.67 ± 5.50	0.67
Body fat rate, %	33.62 ± 7.53	31.42 ± 6.69	0.23
Visceral fat area, cm^2^	124.05 ± 42.52	112.00 ± 29.72	0.21
UA, μmol/L	487.82 ± 88.69	484.44 ± 70.75	0.87
GLU, mmol/L	5.12 ± 0.60	5.64 ± 1.00	**0.01**
TC, mmol/L	5.04 ± 1.04	4.88 ± 0.90	0.52
TG, mmol/L*	1.575 (1.235, 2.590)	1.570 (1.145, 2.480)	0.19
HDLC, mmol/L	1.20 ± 0.21	1.15 ± 0.16	0.29
LDLC, mmol/L	3.11 ± 0.87	3.08 ± 0.68	0.89
WBC, 109/L	7.16 ± 1.71	7.26 ± 1.21	0.79
ESR, mm/h*	6.000 (3.000, 11.500)	5.500 (2.000, 8.750)	0.25
XOD, ng/mL*	3.483 (1.935, 4.369)	2.276 (1.519, 3.457)	**<0.01**
Leptin, pg./mL	5077.02 ± 3027.44	2986.90 ± 1952.31	**<0.01**
ADI, ug/mL*	2.054 (1.041, 2.263)	1.656 (1.334, 2.323)	0.11
IL1, pg./mL*	3.574 (0.934, 9.100)	3.662 (0.793, 5.176)	0.23
IL2, pg./mL*	4.110 (3.360, 10.440)	4.040 (3.375, 10.705)	0.81
IL4, pg./mL*	3.630 (2.273, 5.320)	3.600 (2.390, 4.575)	0.37
IL6, pg./mL*	3.370 (2.598, 5.355)	4.090 (2.885, 6.110)	0.39
IL10, pg./mL*	3.915 (2.900, 6.375)	3.830 (2.850, 6.400)	0.71
TNFα, pg./mL*	3.525 (2.588, 4.803)	3.670 (2.730, 4.290)	0.13

Values are presented as means ± SDs, medians (interquartile ranges), or numbers (%). Comparisons between intervention and control groups were performed using the independent-sample *t*-test, Mann–Whitney U-test or chi-squared test. *p* values less than 0.05 are bolded.

BMI, body mass index; UA, uric acid; GLU, glucose; TC, total cholesterol; TG, triglyceride; HDLC, high-density lipoprotein cholesterol; LDLC, low-density lipoprotein cholesterol; WBC, white blood cell; ESR, erythrocyte sedimentation rate; XOD, xanthine oxidase; ADI, adiponectin; IL, interleukin; TNFα, Tumor necrosis factor α.

* Values were analyzed using the Mann–Whitney U-test because these variables did not exhibit normality.

### Primary and secondary endpoints

Finally, a total of 49 participants in the intervention group and 25 participants in the control group completed the trial with satisfactory dietary compliance, despite some loss to follow-up at various stages of the study. No adverse events were reported throughout the entire duration of the trial. Significant reductions in body fat rate and UA levels were observed after 3 months in both groups (*P* all <0.001 in the intervention group and *p* = 0.01 and 0.046 separately in the control group). In addition, notable decreases in visceral fat area, total cholesterol, triglyceride and three interleukins— IL2, IL4 and IL10— were recorded specifically within the intervention group (*p* = 0.001, 0.01, 0.04, 0.001, 0.02 and 0.03 separately). Conversely, an increase in XOD activity was significantly noted at month three within the control group (*p* = 0.01). When comparing outcomes between the two groups, significant differences were identified regarding UA levels, visceral fat area, and XOD activity (Table 2).

**Table 2:** Comparisons between intervention group and control groups.

Variables	Intervention group (*n* = 49)		Control group (*n* = 25)		Change difference between Groups		
	Change at month 3^#^	*p*-value	Change at month 3^#^	*p*-value	t value/Z value	*p*-value	95% confidence intervals
Body fat rate, %	0.90 ± 1.39	**<0.001**	0.70 ± 1.24	**0.01**	0.60	0.55	−0.46, 0.86
Visceral fat area, cm^2^	4.74 ± 7.37	**<0.001**	−0.36 ± 4.29	0.66	3.38	**0.001**	2.10, 8.10
UA, μmol/L	55.00 ± 62.39	**<0.001**	25.00 ± 59.52	**0.046**	2.02	**0.049**	0.14, 59.86
GLU, mmol/L*	−0.08 (−0.37, 0.18)	0.55	0.24 (−0.20, 0.89)	0.28	−1.42	0.16	−0.54, 0.07
TC, mmol/L	0.23 ± 0.55	**0.01**	0.09 ± 0.43	0.32	1.13	0.26	−0.11, 0.39
TG, mmol/L*	0.22 (−0.17, 0.48)	**0.03**	0.17 (−0.12, 0.36)	0.22	−0.59	0.56	−0.18, 0.32
HDLC, mmol/L	−0.01 ± 0.15	0.61	−0.01 ± 0.09	0.53	0.04	0.97	−0.06, 0.07
LDLC, mmol/L	0.13 ± 0.50	0.08	0.08 ± 0.33	0.24	0.45	0.66	−0.17, 0.27
WBC, 109/L	0.07 ± 1.56	0.77	−0.21 ± 1.13	0.37	0.30	0.76	−0.69, 0.93
ESR, mm/h*	0.00 (−3.50, 2.00)	0.50	0.00 (−1.50, 0.00)	0.10	−0.77	0.44	−1.00, 2.00
XOD, ng/mL*	0.07 (−1.65, 0.99)	0.67	−0.33 (−2.23, 0.07)	**0.01**	−2.25	**0.03**	0.09, 1.85
Leptin, pg./mL	−378.46 ± 2981.77	0.44	−950.99 ± 2351.50	0.08	1.70	0.09	−265.71, 3384.98
ADI, ug/mL*	−0.08 (0.55, 0.56)	0.99	0.42 (−0.31, 0.94)	0.10	−1.47	0.14	−0.82, 0.11
IL1, pg./mL*	0.42 (−1.89, 5.08)	0.07	0.73 (−2.19, 1.59)	0.74	−0.63	0.53	−1.05, 3.23
IL2, pg./mL*	0.78 (0.05, 2.96)	**<0.001**	0.53 (−0.40, 3.52)	0.07	−0.49	0.62	−0.74, 1.36
IL4, pg./mL*	0.70 (−0.72, 2.86)	**0.02**	0.39 (−0.46, 0.89)	0.63	−1.05	0.30	−0.67, 2.03
IL6, pg./mL*	0.47 (−0.98, 1.84)	0.26	0.39 (−0.46, 1.76)	0.35	−0.18	0.86	−1.36, 1.36
IL10, pg./mL*	0.24 (−0.39, 1.99)	**0.03**	0.40 (−0.34, 1.41)	0.13	−0.12	0.90	−0.97, 1.11
TNFα, pg./mL*	0.66 (−0.85, 2.00)	0.08	0.19 (−1.55, 1.37)	0.62	−0.69	0.49	−0.64, 1.60

Within-group *p*-values were obtained using paired *t*-tests or the Wilcoxon signed rank test. Differences at 3 months were compared between the intervention and control groups using the independent-sample *t*-test or the Mann–Whitney U-test, then presented as means or medians (interquartile ranges) to represent mean changes at 3 months.

BMI, body mass index; UA, uric acid; GLU, glucose; TC, total cholesterol; TG, triglyceride; HDLC, high-density lipoprotein cholesterol; LDLC, low-density lipoprotein cholesterol; WBC, white blood cell; ESR, erythrocyte sedimentation rate; XOD, xanthine oxidase; ADI, adiponectin; IL, interleukin; TNFα, Tumor necrosis factor α. *p* values less than 0.05 are bolded.

^#^ Various were analyzed over a 3-month period from the baseline to the end of the trial, and the data were calculated as the difference obtained by subtracting the value after the intervention from that before the intervention.

* Values were analyzed using the Wilcoxon signed rank test and Mann–Whitney U-test because these variables did not exhibit normality.

**Figure 2:**
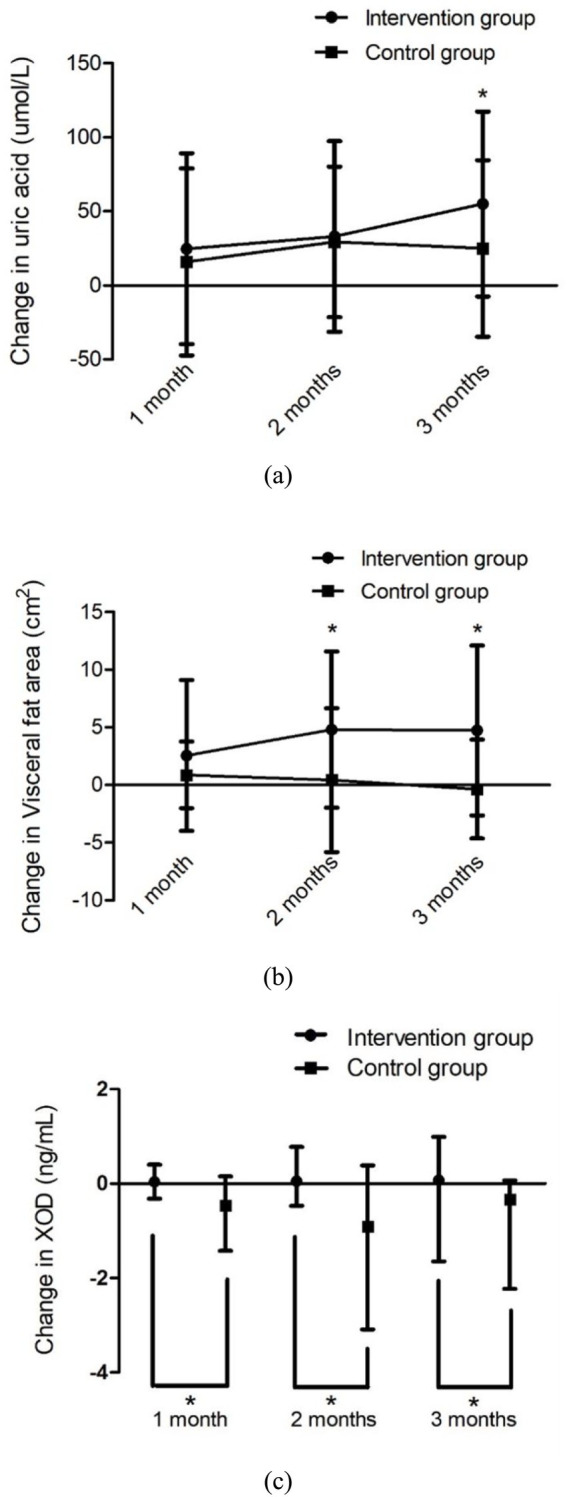
Changes in UA level, visceral fat area and XOD during the 3-month study. Values of UA and visceral fat area are presented as mean ± SD, and values of XOD are presented as median (interquartile ranges). **(a)** Change in UA level over time, **(b)** change in visceral fat area over time, and **(c)** change in XOD over time. * *p* < 0.005 vs. each control group.

The changes in UA levels, visceral fat area and XOD activity across each group over time are illustrated in Figure 2. A significant difference was observed in the change of UA levels between the two groups at three-month post-enrollment (*t* = 2.02, *p* = 0.049). Additionally, a notable difference in the change of visceral fat area between the two groups was detected at both the second month (4.80 ± 6.77 for the intervention group versus 0.42 ± 6.24 for the control group; *t* = 2.66, *p* = 0.01, 95%CI [1.10, 7.68]) and third months (*t* = 3.38, *p* = 0.001) following enrollment. Furthermore, significant differences in XOD activity changes were identified at multiple time points: during the first month (0.03 (−0.29, 0.40) for the intervention group versus −0.46 (−1.42, 0.15) for the control group; *Z* = −2.09, *p* = 0.04, 95%CI [0.07, 1.31]), second month (0.06 (−0.47, 0.78) for the intervention group versus −1.00 (−3.09, 0.17) for the control group; *Z* = −2.49, *p* = 0.01, 95%CI [0.25, 3.02]), and third month (*Z* = −2.25, *p* = 0.03) after enrollment.

### Analysis of the influencing factors of UA variation

Correlation analyses were conducted to elucidate both the baseline characteristics and the changes in clinical and laboratory parameters associated with UA reduction within each group (Table 3). In the intervention group, a higher baseline level of UA, a lower baseline level of leptin, and more pronounced changes in TC and LDLC were significantly correlated with UA reduction over a three-month period. Conversely, in the control group, greater fluctuations in WBC were significantly associated with UA decline at 3 months.

**Table 3:** Relationships of change in UA with baseline and changes of clinical and laboratory parameters.

Variables	Intervention group (*n* = 49)		Control group (*n* = 25)	
	Correlation coefficient	*p*-value	Correlation coefficient	*p*-value
Age, years	−0.21*	0.32	0.18*	0.39
Sex	−0.25*	0.08	0.08*	0.69
Weight, kg	0.03	0.84	−0.06	0.78
Change in weight, kg	0.23*	0.13	0.06	0.77
BMI, kg/m^2^	−0.04	0.78	0.03	0.89
Change in BMI, kg/m^2^	0.23	0.12	0.12*	0.57
Body fat rate, %	−0.25	0.09	0.09	0.67
Change in Body fat rate, %	0.25	0.10	0.13	0.53
Visceral fat area, cm^2^	−0.12	0.40	0.22*	0.29
Change in Visceral fat area, cm^2^	0.18*	0.21	−0.08*	0.69
UA, μmol/L	0.55	**<0.001**	0.08	0.71
GLU, mmol/L	−0.28	0.06	0.02	0.92
Change in GLU, mmol/L	−0.002	0.99	−0.21	0.33
TC, mmol/L	0.23	0.12	−0.03	0.88
Change in TC, mmol/L	0.36	**0.01**	−0.20	0.35
TG, mmol/L	−0.03*	0.80	0.04	0.84
Change in TG, mmol/L	0.01	0.93	0.12	0.59
HDLC, mmol/L	−0.14	0.36	0.09	0.67
Change in HDLC, mmol/L	−0.05	0.74	−0.01	0.97
LDLC, mmol/L	0.23	0.11	−0.11	0.62
Change in LDLC, mmol/L	0.31	**0.03**	−0.30	0.14
WBC, 109/L	0.03	0.82	0.07	0.75
Change in WBC, 10^9^/L	0.22	0.14	0.46	**0.02**
ESR, mm/h	−0.24*	0.10	−0.10*	0.63
Change in ESR, mm/h	−0.07*	0.65	0.18*	0.40
XOD, ng/mL	0.14*	0.14	0.15	0.49
Change in XOD, ng/mL	0.18	0.23	0.05	0.81
Leptin, pg./mL	−0.33	**0.02**	0.02	0.92
Change in Leptin, pg./mL	0.03	0.87	−0.14	0.51
ADI, ug/mL	0.24	0.10	0.01*	0.98
Change in ADI, ug/mL	0.01	0.98	0.25	0.24
IL1, pg./mL	−0.03*	0.82	−0.003	0.99
Change in IL1, pg./mL	−0.18	0.21	0.08	0.70
IL2, pg./mL	0.19*	0.19	−0.10*	0.66
Change in IL2, pg./mL	0.19*	0.19	−0.11*	0.62
IL4, pg./mL	0.19*	0.24	−0.39	0.10
Change in IL4, pg./mL	0.24*	0.24	−0.33	0.24
IL6, pg./mL	0.20*	0.21	−0.28	0.25
Change in IL6, pg./mL	0.21*	0.21	−0.45	0.08
IL10, pg./mL	0.21*	0.82	−0.10	0.70
Change in IL10, pg./mL	0.12*	0.12	0.10	0.72
TNFα, pg./mL	0.12*	0.62	−0.11	0.62
Change in TNFα, pg./mL	0.19*	0.21	−0.21	0.33

* *p*-values were obtained by Spearman rank correlation analysis.

*p*-values without * were obtained by Pearson rank correlation analysis. *p* values less than 0.05 are bolded.

Multiple linear regression analysis in the intervention group (adjusted for baseline level of UA, leptin and XOD, and 3-months’ changes in TC, LDLC and weight) revealed that high baseline UA and XOD activity, low baseline leptin, and significant decline in TC and weight were independently associated with UA decline after 3 months of intervention (Table 4).

**Table 4:** The influencing factors of the change level of UA in the intervention group, determined by multiple linear regression.

Variables	Standardized regression coefficient	*p*-value	95% confidence interval
UA, μmol/L	0.381	**0.010**	0.067, 0.470
Leptin, pg./mL	−0.276	**0.049**	−0.009, 0.000
Change in TC, mmol/L	0.659	**0.012**	18.244, 140.126
Change in LDLC, mmol/L	−0.512	0.050	−131.917, 0.041
XOD, ng/mL	0.371	**0.003**	2.828, 13.121
Change in weight, kg	0.339	**0.015**	1.260, 10.950

*p*-values less than 0.05 are bolded.

## Discussion

Medicinal plants are key sources of functional foods and pharmacologically active compounds. Barley grass, obtained from the young shoots of *Hordeum vulgare* L. (Poaceae family), originated in the Fertile Crescent approximately 11,000 years ago and has long been used in traditional medicine. Several studies have investigated its chemical composition (7). The most comprehensive analysis to date, conducted by Beijing Anzhen Hospital, identified 273 nutrients, including chlorophyll, flavonoids, over 100 enzymes, 18 amino acids, more than 20 vitamins, 70 minerals, hexacosanol P4D1 enzyme, and soluble fiber. Barley green represents a nutritionally dense compound with potential functional health benefits.

Barley green has demonstrated anti-inflammatory, antioxidant, and glucose- and lipid-regulating properties in various studies (6). In alignment with the findings from the aforementioned program in the sample size calculation section (Shen F, et al., J Mod Med Health, October 2021, Vol.37, No.20. DOI: 10.3969/j.issn.1009-5519.2021.20.012), our study also revealed that after 3 months of intervention, the reduction in UA levels within the intervention group was significantly greater than that in the control group (55.00 ± 62.39 versus 25.00 ± 59.52; *p* = 0.049). This supports effectiveness of barley green in lowering blood UA levels in patients with hyperuricemia. A Japanese study of 111 participants also found a 0.21 ± 0.56 mg/dL decrease in UA levels among those taking fermented barley extract P (FBEP), further indicating its efficacy and safety for mild hyperuricemia (8).

Studies on the mechanism by which barley green regulates UA metabolism have mainly focused on its individual nutrients and alkalinity. It has a high alkalinity of 66.4 and contain abundant chlorophyll, plant flavonoids, and active enzymes like superoxide dismutase (SOD), which act as potent antioxidants to regulate purine metabolism and uric acid production (9). Their flavonoids may help manage gouty arthritis by suppressing inflammatory mediator release from neutrophils and reducing cytokine secretion and gene expression triggered by uric acid crystals (10). They may also prevent hyperuricemia by inhibiting XOD activity and enhancing uric acid excretion. XOD is the rate-limiting enzyme in purine metabolism, catalyzing the conversion of hypoxanthine to xanthine and then to uric acid. Its activity directly determines uric acid production. Studies in animals, cell models, and human samples show that increased XOD activity leads to higher serum uric acid levels, while XOD inhibition—such as by allopurinol or febuxostat—lowers uric acid (11). Emerging evidence suggests that several natural XOIs can effectively lower uric acid levels by inhibiting XOD activity. Potent N-heterocyclic analogues, dietary flavonoids, and ellagic acid function as low-micromolar, reversible, mixed-type XOIs. These compounds suppress hepatic XOD activity by 30–50% and normalize plasma uric acid in hyperuricemic mice, demonstrating efficacy comparable to that of allopurinol (12–14). This further validates XOD as a key therapeutic target for anti hyperuricemic intervention.

Recent studies have explored food-derived XOIs (15). Qi Xin et al. evaluated the XOD inhibitory effects of 29 common vegetable extracts. At 200 mg/mL, 20 extracts showed inhibition, with three exceeding 50%. At 100 mg/mL, 17 extracts were active, one surpassing 50%. At 50 mg/mL, 14 extracts showed inhibition, again with one above 50%. At 25 mg/mL, 11 extracts had some effect, but none exceeded 50%. The most potent inhibitors were ginger, garlic, and yam, with IC50 values of 82.7 μg/mL, 86.2 μg/mL, and 142.3 μg/mL, respectively. A Malaysia study (16) also found that *Acorus gramineus* and Melastoma species may lower UA levels through flavonoid compounds and phenylethanoid glycosides via XOD inhibition. Globally, however, research on how barley green affects serum UA levels in humans remains limited. Our study found that after a three-month trial, the intervention group showed a significantly greater decrease in XOD activity than the control group. Although no significant within-group changes were observed over time, the trend suggested a progressive decline in XOD activity during treatment. These findings indicate that barley green may act as a novel plant-based XOI with potential for lowering UA levels in humans.

The findings of regression analysis revealed that greater reductions in UA levels after 3 months of barley green intake were associated with higher baseline UA and XOD levels, lower initial leptin levels, and larger fluctuations in TC, LDLC, and weight during the intervention. Notably, smaller increases in leptin levels during the intervention were linked to more pronounced UA reductions. UA metabolism is influenced not only by XOD activity but also by leptin levels, a protein hormone predominantly produced by adipose tissue (17). Studies suggest that leptin can affect UA levels by regulating its excretion (18), and the Olivetti Heart Study (OHS) (19) indicated a positive correlation between circulating leptin levels and UA, independent of potential confounding factors. Our findings indicate that barley green may have a stronger UA-lowering effect in individuals with minimal increases in circulating leptin levels. Furthermore, numerous studies have confirmed a close link between lipid and UA metabolism (20, 21). Previous evidence shows barley green can reduce plasma lipids in hyperlipidemia patients (5), and our study supports this by showing that greater decreases in TC or LDLC correlate with larger UA reductions. Consistent with earlier findings (22, 23), we also confirmed a relationship between body weight and UA levels.

This trial also found that the intervention group, which consumed barley green, experienced a significant decrease in visceral fat over time compared to the control group. While body weight and BMI are widely studied, fewer studies have focused on the relationship between body composition—especially visceral fat—and UA metabolism. A cross-sectional study (24) involving 121 healthy obese individuals and 99 lean subjects showed that fat mass (%FM) and fat-free mass (FFM) were significant predictors of serum UA levels (*p* < 0.0001). Another study on metabolic dysfunction associated with fatty liver disease (MAFLD) found that weight loss—especially the reduction of visceral fat—plays a crucial role in decreasing serum UA levels and managing the condition (22). In this trial, after 1, 2, and 3 months of barley green intake, the intervention group showed significantly lower UA levels and a notable decrease in visceral fat compared to the control group, aligning with previous findings. These results suggest that barley green may help reduce visceral fat in hyperuricemia patients, offering new insights into UA-lowering mechanism.

Despite these promising findings, this research has limitations. Firstly, no significant decrease in XOD activity was observed within the intervention group, although a statistically significant difference was found between the intervention and control groups. In addition to limitations in XOD activity detection methods and sample preservation and transportation conditions, further experiments—such as *in vitro* cell studies and animal trials—are needed to confirm these results. The lack of blinding may introduce performance and detection biases. Although a pre-post difference model (*Δ* = pre − post) was used to remove subject-specific means, baseline imbalances in glucose, XOD, and leptin could still interact with the intervention, leading to regression to the mean—particularly in participants with higher initial values. This may exaggerate the observed decline in XOD activity and increase type I error risk. Another limitation is the use of listwise deletion, which may cause selection bias if missing data are associated with predictors or outcomes. The small sample size also limited the precision of the results. The short study duration and lack of long-term follow-up further restrict the generalizability of the findings. Given known sex differences in UA metabolism, future studies should include sex-based subgroup analyses. Due to reasons including individual differences, the confidence intervals of a few specific variables’ statistical values are relatively wide or the standard deviations are relatively large, which affects the reliability of the results. This issue needs to be deeply explored in future experiments to identify the underlying causes and minimize its impact on data reliability. Finally, potential funding bias suggests that the results should be interpreted with caution.

Therefore, further verification experiments on XOD activity are urgently needed. Future studies should focus on improving the experimental design by using randomization and blinding, and on refining statistical methods using mixed-effects models and appropriate techniques for handling missing data. Expanding the sample size, extending the trial duration, and conducting long-term follow-up investigations are also essential to enhance the reliability and generalizability of the results.

## Conclusion

The oral administration of barley green supplements, alongside a balanced diet, demonstrated a beneficial effect on UA control, as well as a reduction in XOD activity, and visceral fat area. The reduction in XOD activity may represent one of the mechanisms through which barley green exerts its UA-lowering effects. Baseline UA levels, XOD activity, and leptin concentrations, along with changes in TC and body weight, may serve as independent predictive factors for UA decline.

## Data Availability

The raw data supporting the conclusions of this article will be made available by the authors, without undue reservation.

## Footnotes

^1^ http://dg.cnsoc.org/upload/affix/20220426154943388.jpg
