# Supplementary figures and images for: Effects of barley green on uric acid, inflammatory factors, xanthine oxidase activity and body composition of patients with hyperuricemia: a randomized controlled trial
Source: Front Nutr. 2025 Oct 31;12:1684829. doi: 10.3389/fnut.2025.1684829 (PMC12616856; doi:10.3389/fnut.2025.1684829)

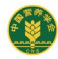

## 中国居民平衡膳食宝塔(2022)

Chinese Food Guide Pagoda(2022)

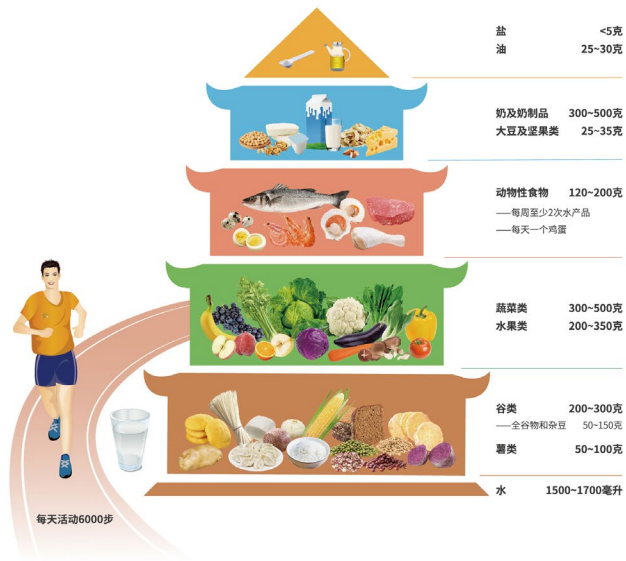

(<http://dg.cnsoc.org/upload/affix/20220426154943388.jpg>)

Supplement: Supplementary file 1 [file Image_1.pdf]
